# Processing two-dimensional X-ray diffraction and small-angle scattering data in *DAWN 2*

**DOI:** 10.1107/S1600576717004708

**Published:** 2017-05-08

**Authors:** J. Filik, A. W. Ashton, P. C. Y. Chang, P. A. Chater, S. J. Day, M. Drakopoulos, M. W. Gerring, M. L. Hart, O. V. Magdysyuk, S. Michalik, A. Smith, C. C. Tang, N. J. Terrill, M. T. Wharmby, H. Wilhelm

**Affiliations:** a Diamond Light Source Ltd, Harwell Science and Innovation Campus, Didcot OX11 0DE, UK

**Keywords:** X-ray diffraction, data processing, computer programs

## Abstract

A software package for the calibration and processing of powder X-ray diffraction and small-angle X-ray scattering data is presented. It provides a multitude of data processing and visualization tools as well as a command-line scripting interface for on-the-fly processing and the incorporation of complex data treatment tasks. Customizable processing chains permit the execution of many data processing steps to convert a single image or a batch of raw two-dimensional data into meaningful data and one-dimensional diffractograms. The processed data files contain the full data provenance of each process applied to the data. The calibration routines can run automatically even for high energies and also for large detector tilt angles. Some of the functionalities are highlighted by specific use cases.

## Abstract 2

The *Powder Calibration* and *Processing* packages implemented in *DAWN 2* provide an automated diffraction-geometry calibration and data processing environment for two-dimensional diffraction experiments. The customizable processing chains permit the execution of data processing steps to convert raw two-dimensional data into meaningful data and diffractograms. The provenance of the processed data is maintained, which guarantees reproducibility and transparency of the data treatment.

## 1. Introduction   

Experiments on powder X-ray diffraction (PXRD) and small-angle X-ray scattering (SAXS) instruments at synchrotron beamlines are becoming ever more automated and consequently produce an increasing amount of raw data. Moreover, modern area detectors can acquire two-dimensional (2D) data sets at frame rates of the order of 1 ms, resulting in an overwhelming number of raw images. These circumstances demand an easy-to-use and flexible data processing software package for use both during and after the data collection.

Among the large number of software packages that can deal with two-dimensional data sets, *Fit2D* (Hammersley *et al.*, 1996 ▸; Hammersley, 2016 ▸) is the most well known. More recent software developments can be proprietary (*Datasqueeze 3.0*; Heiney, 2005 ▸) or open source but written in expensive commercial languages (*Nika*; Ilavsky, 2012 ▸). In contrast to this are open-source developments using freely available languages, primarily Python [*GSAS-II* (Toby & Von Dreele, 2013 ▸), *DPDAK* (Benecke *et al.*, 2014 ▸), *pyFAI* (Ashiotis *et al.*, 2015 ▸) and *DIOPTAS* (Prescher & Prakapenka, 2015 ▸)]. Packages are often targeted at a particular experiment (*DIOPTAS* for PXRD, *DPDAK* for SAXS) and some are based on an extensible architecture (*DPDAK*) to provide flexibility.

The *Data Analysis Workbench* (*DAWN*) developed at the Diamond Light Source (DLS) originally had a limited ability for processing PXRD or SAXS data (Basham *et al.*, 2015 ▸). However, *DAWN* has been substantially rewritten, and *DAWN 2* now provides a versatile data analysis platform for additional software developments. The most recently added tools are specific for the automated calibration and the batch processing of 2D-PXRD and SAXS diffraction data. *DAWN 2* is free, open source and available for anyone to analyse two-dimensional scattering data.^1^

The key points of the calibration and processing packages are (i) a new user interface to build custom data processing pipelines for a step-by-step analysis or a batch processing of the raw data sets; (ii) a command-line version of *DAWN 2* and a scripting interface to the calibration and reduction; (iii) full data provenance of the processed data, *i.e.* the ability to automatically save all processing steps and parameters that are needed to recreate the same output; (iv) standardized and versatile data input and output; and (v) an implementation of a calibration routine which provides accurate values for energy and the detector geometry even for experiments at very high energy (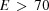
 keV) or large detector tilts.

Although these features are a relatively recent addition they have already been used for many experiments (*e.g.* Clout *et al.*, 2016 ▸; Collins *et al.*, 2015 ▸; Michalik *et al.*, 2016 ▸; Pethes *et al.*, 2016 ▸; Pollastri *et al.*, 2016 ▸; Sanchez-Fernandez *et al.*, 2016 ▸; Scott *et al.*, 2016 ▸; Murray *et al.*, 2017 ▸). In this article some details of the new calibration routines (§2) and the data processing functionality (§3) will be presented. Finally, several use cases (§4) highlight how some generic features are deployed on different types of experiments.

## 2. Calibration of the diffraction geometry   

The geometry of a diffraction experiment using a two-dimensional detector is given by six independent parameters: the distance between the sample and detector, two orthogonal tilt angles of the detector, two orthogonal detector coordinates for the position of the beam centre (which can be inside or outside of the detector surface), and the energy or wavelength of the X-rays. Throughout this paper we will refer to these six parameters as the diffraction geometry.

**Figure 1:**
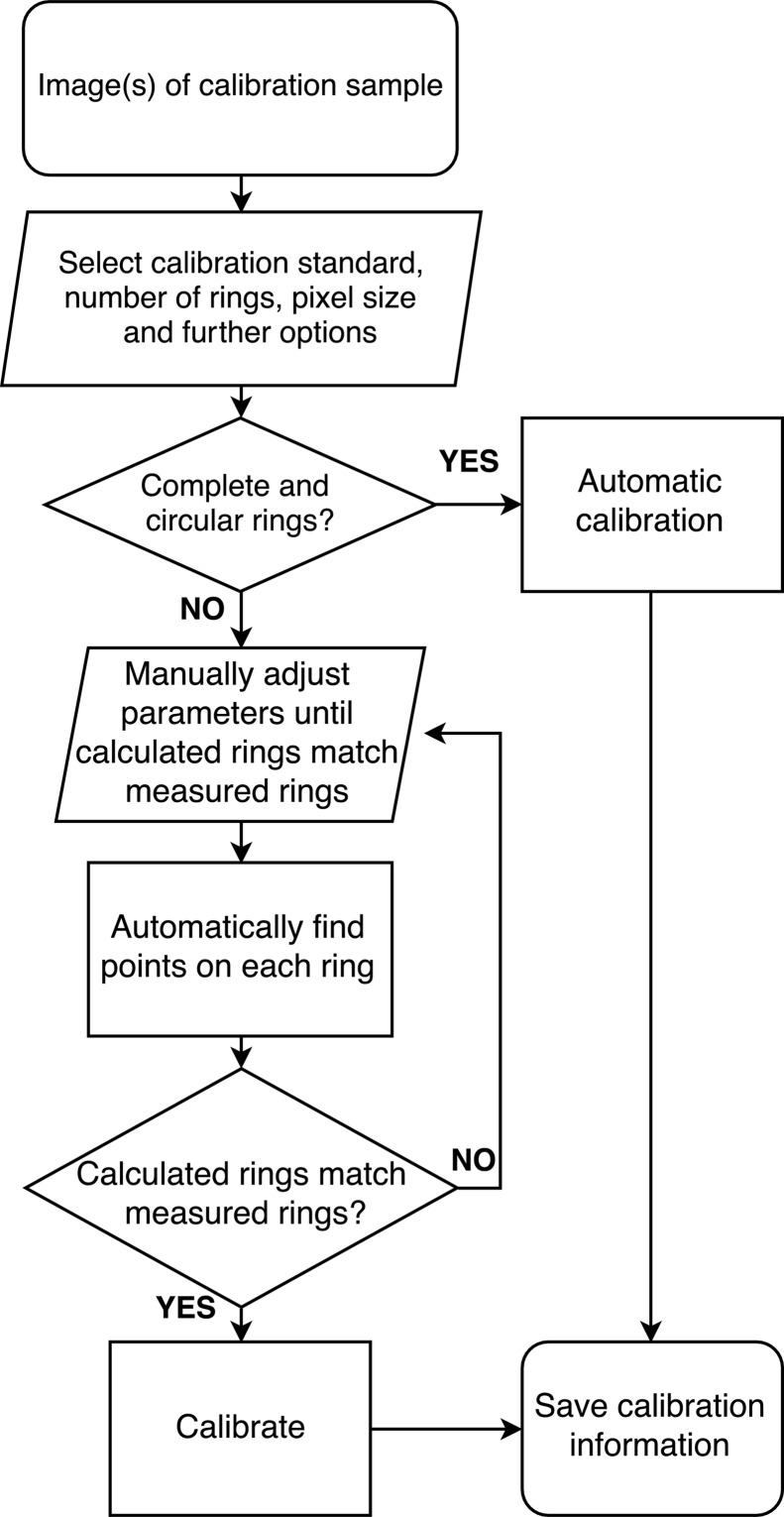
Work flow for obtaining the parameters of the diffraction geometry. After a (set of) two-dimensional calibration image(s) has been loaded and the process has been initialized, the details of the data define how to proceed. Data with almost complete Debye–Scherrer rings of low ellipticity are suited for the automated process. Highly elliptical or partial rings require a manual treatment. Ultimately the calibration information is saved and made available for the data processing.

A general calibration routine for PXRD and SAXS experiments has to fulfil many different requirements. It has to (i) reliably determine the diffraction geometry, (ii) be able to execute the routine automatically, and (iii) support nonstandard geometries with the beam centre off the detector or a detector at large tilt angles. To meet these different requirements, the *Powder Calibration* perspective in *DAWN* contains separate routines for calibrating the diffraction geometries of PXRD and SAXS experiments with an appropriate calibration sample (see Fig. 1 ▸): (i) a fully automatic routine, which uses either a single image or multiple images for standard detector configurations and (ii) a manual routine which is suitable for detector orientations that result in highly elliptical rings or a beam centre outside the detector frame. At the end of either calibration routine, the determined parameters of the diffraction geometry are saved as a standard NeXus file (Könnecke *et al.*, 2015 ▸), which also stores the provenance of the calibration, *i.e.* other relevant information such as the used calibration standard and the details of the selected routine.

The core of the automatic calibration routine implemented in *DAWN* is a non-iterative, algebraic method originally developed by Hart *et al.* (2013 ▸). It uses multiple two-dimensional diffraction patterns of a calibration standard, taken at a precisely known set of increments of the sample-to-detector distance, and determines the calibration geometry from ellipses fitted to all Debye–Scherrer rings simultaneously. Alternatively, the routine allows all parameters of the diffraction geometry to be determined from a single two-dimensional diffraction pattern, if sufficient high-angle reflections are present. However, if a precise value for the X-ray energy is already known, for example from a scan through an absorption edge, the routine requires only one two-dimensional diffraction image of a calibration standard at a single distance.

The calibration routine starts by automatically determining the approximate radius of each ring assuming a position of the direct X-ray beam on the detector. Thus, it is only applicable to small tilt angles of the detector and with the beam centre on the detector. Initially, each ring is matched against an azimuthally integrated pattern. The rings are then located precisely by taking several hundred line profiles across each circumference and fitting a Gaussian peak shape to each of them. This results in a set of points of maximum intensity for each diffraction ring. Fitting an ellipse to each of these sets of points yields ellipse centres and semi-major axes. The output of these fits is then used to determine the parameters of the diffraction geometry. Uncertainty estimates for these parameters can be generated by using this output as input to the least-squares routine that is part of the manual calibration.

The manual calibration routine is used for detectors at large tilt angles or images that contain only arcs of Debye–Scherrer rings. In this case initial guess values for the parameters of the diffraction geometry are required. Then the diffraction rings are calculated and overlaid with the measured diffraction pattern. If the data and the calculated diffraction rings match then the calculated reflection positions are used in the ring-finding routine described above. The points found on each ring are then used in a least-squares optimization routine to determine the parameters of the diffraction geometry and their uncertainties. If the ring-finding routine fails, the initial guess values need to be adjusted before re-running the routine.

**Figure 2:**
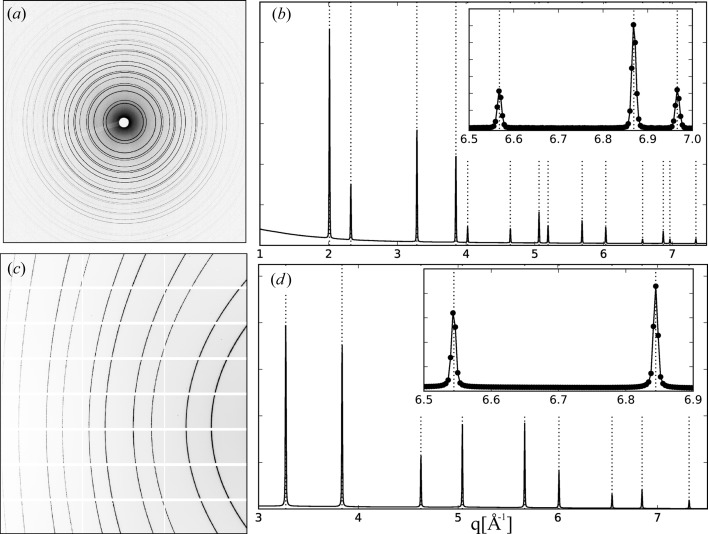
(*a*) Powder pattern of the calibration standard CeO_2_ (NIST, SRM674b) recorded at beamline I11 with a Pixium area detector (RF4343, Thales Electron Devices S. A.) using an X-ray energy of 25.5 keV. (*b*) The one-dimensional diffractogram, intensity *versus q*, of the azimuthally integrated two-dimensional pattern shown in (*a*). Superimposed are dotted lines which indicate the *q* values of the calibration standard. The inset shows a zoom into the highest-*q* region and highlights the quality of the calibration. (*c*) Powder pattern of Si (NIST, SRM640c) recorded with X-rays of 12 keV at beamline I16 with a Pilatus 2M detector (Dectris Ltd) at a tilt angle of 45° degree. The white grid reflects the tiling of the detector. (*d*) The integrated pattern of (*c*) shows that all peak positions are in perfect agreement with the calculated *q* values of Si (dotted lines). This is emphasized by the zoom in to the high-*q* data shown in the inset.

The application of these two cases is exemplified in Fig. 2 ▸. A typical powder diffraction pattern that consists of a large number of nearly circular Debye–Scherrer rings centred roughly in the middle of an area detector is shown in Fig. 2 ▸(*a*). In this case the automatic calibration routine produces the integrated powder pattern of the calibration sample shown as intensity *versus q* in Fig. 2 ▸(*b*). It is in excellent agreement [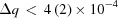
 Å^−1^] with the calculated *q* values of the calibrant (vertical dotted lines) over the entire accessible *q* range. The partial, highly elliptical diffraction rings of a calibration sample recorded with a significantly tilted area detector are depicted in Fig. 2 ▸(*c*). Here the reduction to a one-dimensional data set requires a manual adjustment of a calculated ring pattern to the partial rings. Once the calculated rings match the measured ones, the calibration routine is able to produce a very good agreement of the observed and calculated *q* values of the calibrant [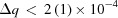
 Å^−1^] as shown in Fig. 2 ▸(*d*).

## 3. Data processing   

In the *DAWN* data processing framework, each individual data processing step is designed as an individual isolated module. A com­plete processing chain is built from a sequence of these modules. This provides a highly flexible environment to accommodate different demands on the data processing.

At the architectural level, each module consists of two classes: the operation, which contains the mathematical algorithm, and a model containing the parameters used by the algorithm, *e.g.* the *q* range to integrate over. The latter can be configured by the user. *DAWN* automatically generates a user interface for the model, so no interface development is needed to add a new module. This modular design is a feature of the Java Eclipse RCP framework in which *DAWN 2* is written. It allows new processing steps to be developed and built outside of the *DAWN 2* source code and installed as a new plug-in. In addition, there are modules that allow a processing algorithm for a specific data treatment or new processing steps to be written as a Python script (Appendix *A* [App appa]).

**Figure 3:**
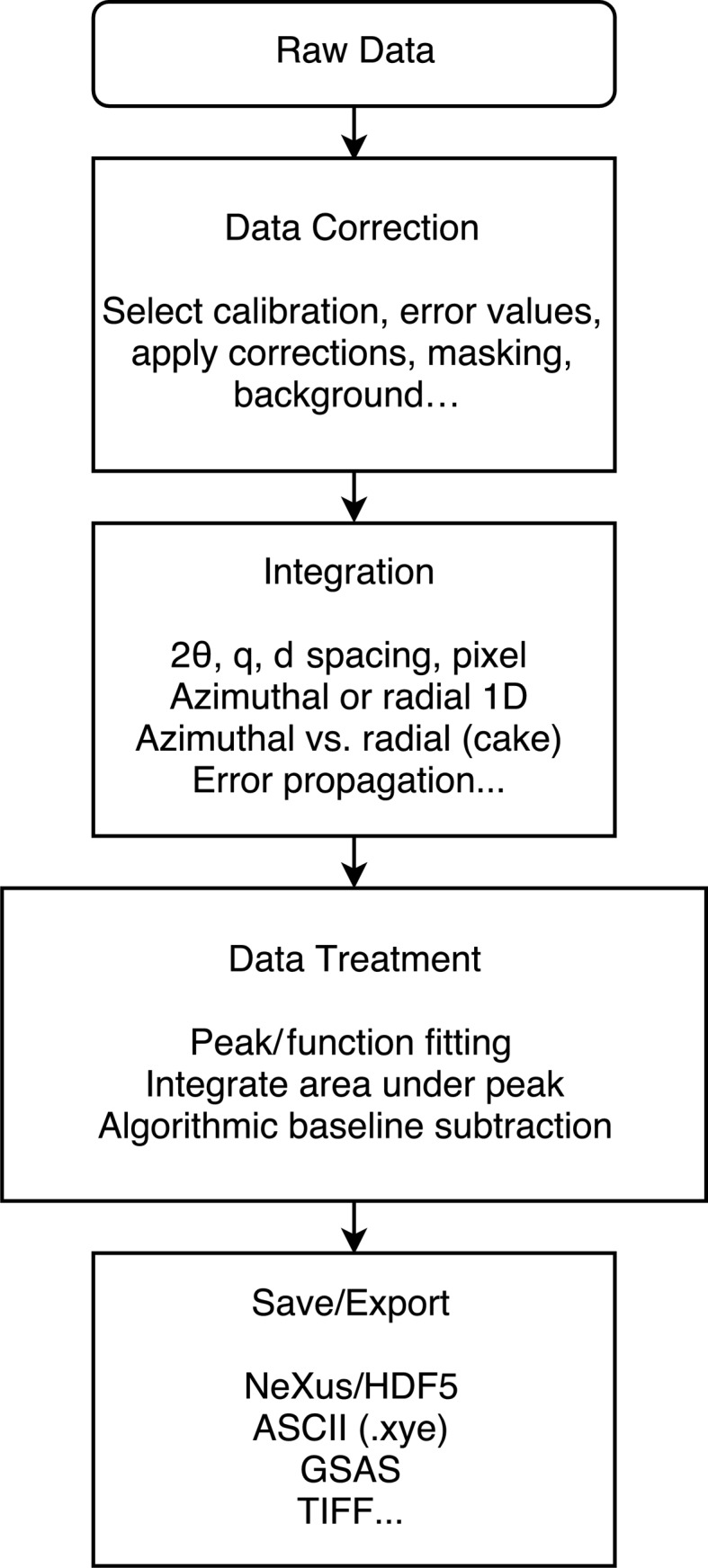
Generic work flow for processing 2D-PXRD and SAXS data. Each task can contain several sub-tasks such as masking, integration, peak fitting and data export. This process chain could contain other or many more tasks.

Fig. 3 ▸ shows a generic processing work flow grouped by the different tasks and the individual modules each task might include. Data are pushed through this processing chain in a pipeline which uses standard *DAWN* file-reading modules (Basham *et al.*, 2015 ▸) to load the data (Appendix *B* [App appb]) and sequentially send the data down the line. As the code for data loading is decoupled from the rest of *DAWN*, new file loaders can easily be added without modifying the core code. Multiple implementations of this pipeline exist for running through the data: in series, parallel, using a *Passerelle* work flow engine (Brockhauser *et al.*, 2012 ▸) or with a runner for live data processing. The pipeline also maintains the rank of the input data; for example, processing data from a grid scan will produce a three-dimensional data set of integrated data and two-dimensional data sets from any scalars derived from the integrated patterns (see §4.3).

The output of the processed files is written as HDF5 files conforming to the NeXus standard. They store the processed data, associated axes and uncertainties, any selected intermediate data (*e.g.* corrected or normalized two-dimensional images), and the provenance of the data processing. Thus, the output files contain details on which data were processed, the processing chain and which parameters were used for the processing modules. This data provenance makes the data treatment transparent and reproducible as the processing chains can be reloaded from the processed data files.

**Figure 4:**
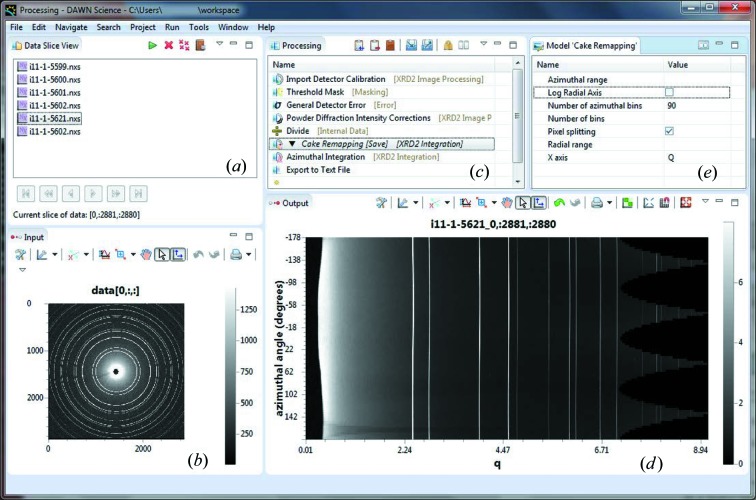
Screenshot of the *Processing* perspective embedded in *DAWN 2*, showing (*a*) the *Data Slice* view with multiple files loaded for batch processing, (*b*) the raw detector data image in the *Input* view, (*c*) the *Processing* view with several processing steps, (*d*) the *Output* view with the processed input data at the selected position in the processing chain (*Cake Remapping*) and (*e*) the *Model* view of the selected *Cake Remapping* processing step with its configurable parameters.

In *DAWN* a processing chain can be built using the *Processing* perspective (see Fig. 4 ▸). The list of files that have been loaded for processing is shown in the *Data Slice* view (Fig. 4 ▸ *a*). Files might be individual images or they may be stacks of images, in which case selecting a file from the list causes the first image from that file to be plotted in the *Input* view (Fig. 4 ▸ *b*). A sequence of processing steps for the data can be built up in the *Processing* view (Fig. 4 ▸ *c*). A process step (module) is added to the processing chain from a drop-down list and its effect on the data is shown immediately in the *Output* view (Fig. 4 ▸ *d*).

Fig. 4 ▸(*c*) shows an example of a processing chain for powder diffraction data. The first module imports the *Detector Calibration*, saved from the *Powder Calibration* perspective. Then a *Threshold Mask* is applied to remove any hot or overexposed pixels from the raw data. Depending on the detector used, the *General Detector Error* module infers the error values for the recorded counts of each pixel in the image. These values will be propagated down the chain. Then the *Powder Diffraction Intensity Corrections* apply a solid angle and polarization correction to the raw image. This is followed by a normalization using *Divide*, which divides the raw intensity values by a reference intensity (*e.g.* 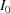 value). The next operation is a *Cake Remapping*, and its result is shown in the *Output* view and saved into the output file (indicated by *[Save]* in the *Processing* view). However, the result is not passed further down the sequence (indicated by the black arrow). This means that the input data to the *Cake Remapping* module ‘pass through’ this step without modification to the *Azimuthal Integration*, which eventually reduces the masked, normalized and intensity-corrected two-dimensional diffraction data to a one-dimensional diffractogram. In the last step this diffractogram is exported to a text file. In this example the output NeXus file contains the cake remapped images and the azimuthally integrated data with intensity errors and any axes selected to propagate with the data (*e.g.* temperature or stage coordinates). Once the processing chain is complete, pressing the run button processes all the images in all the files and yields one output NeXus file for each input file. It is also possible to use *DAWN* from the command line and launch the processing chain to process data files without starting the *Processing* perspective.

When a processing module is selected, such as the *Cake Remapping* in Fig. 4 ▸(*c*), editable parameters associated with the operation are shown in the *Model* view (Fig. 4 ▸ *e*). For this particular example the number of azimuthal bins was specified, a pixel-splitting algorithm was selected and the diffractogram will be plotted *versus q*. Once the model parameters are selected, clicking back on the *Processing* view updates the data shown in the *Output* view up to (and including) the selected step in the process chain. Using the *Processing* view like this allows the result of each intermediate step applied to any image in the *Data Slice* view to be shown in the *Output* view. If the selected file in the *Data Slice* view contains multiple images, the frame-control panel of the *Data Slice* view permits the user to browse individual images. By flicking through the images in this way, the effect of a module or the result of the entire process chain on each image is visualized in the updated *Output* view. These features make it easy to build long complicated processing chains while still being confident that the intermediate steps are being applied correctly.

## 4. Use cases   

In the following we describe how some of the features introduced above are applied to a broad set of experiments at different beamlines at DLS. The benefit of using a processing chain is highlighted by the ‘on-the-fly’ data reduction, which enables an almost real-time monitoring of a solid-state reaction (beamline I12). The automated calibration and the scripting interface to generate one-dimensional diffractograms is exemplified for experiments carried out at the Long-Duration Experiment (LDE) instrument (beamline I11). Finally, the capability to process two-dimensional maps of SAXS data acquired by grid scans (beamline I22) highlights how the rank of the raw data set is maintained.

### 4.1. Calibration at high energy and on-the-fly data reduction   

The diffraction angles at high energies are small, *i.e.* 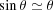. This creates an ambiguity when applying Bragg’s law to a set of Debye–Scherrer rings of different Miller indices when both detector distance and energy are still unknown. The multi-image calibration routine in *DAWN* allows one to overcome this ambiguity and leads to highly accurate and reliable calibration of the diffraction geometry.

*In situ* time-resolved powder diffraction experiments at high energies (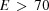
 keV) are challenging and non-routine measurements which may last only a few seconds or up to several hours, potentially producing thousands of images. During these experiments it is important to be able to judge from processed data whether external experimental parameters need to be changed while the experiment is ongoing. For instance, when monitoring crystallization from solution, the crystalline peaks of the product at the initial stage have low intensities and need to be separated from the strong diffuse background. Processing such diffraction patterns therefore requires a series of tasks which need to be carried out on the fly in an automated manner. Such tasks can comprise masking, dynamic background subtraction, normalization and integration of peak intensities.

At beamline I12, the processing chain of *DAWN* was used to process a selection of individual images from the detector as the reaction was happening. Once a chain is saved it can be executed *via* the command line to automatically process data on the fly, *i.e.* data that have just been acquired. Integrating a single image from a Pixium detector (with 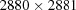 pixels) takes less than 100 ms, and multiple images can be processed at this speed in parallel, making the total processing time essentially dependent on the number of cores on the machine. Any intermediate step in the processing chain can be viewed through the user interface, allowing changes to the masking or background subtraction to be made dynamically, without having to process all the data again.

**Figure 5:**
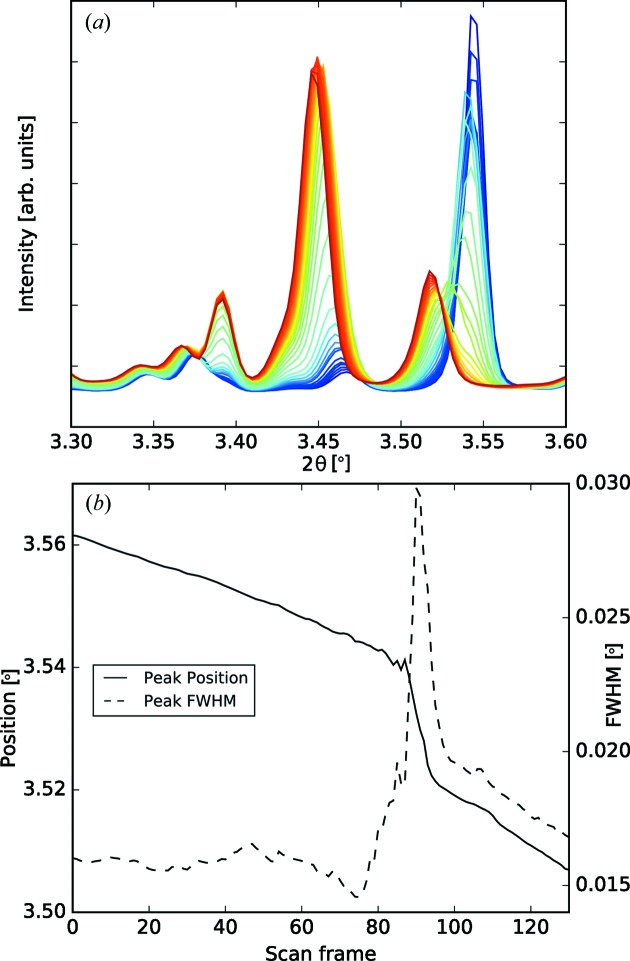
Real-time monitoring of a solid-state transformation. (*a*) A subset of diffractograms around a temperature-induced crystallization process. Only those diffractograms where the reaction took place are shown [from frame 75 (blue) to frame 105 (red)]. (*b*) The evolution of the peak position and its FWHM over the entire measurements indicate at which frame, *i.e.* temperature, the transformation occurred.

Fig. 5 ▸(*a*) shows as an example a set of diffractograms that are the output of a process chain which included a detailed analysis of changes during a solid-state transformation of organic co-crystals. This subset of diffractograms could be easily identified after a peak fitting revealed an obvious change in peak position and full width at half-maximum (FWHM) of a diffraction peak in the region 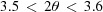
° (Fig. 5 ▸ *b*). When data collections contain thousands of images the benefit of the automatic execution of a processing chain is obvious. Although the individual tasks are quite simple, without an automatic background subtraction and subsequent basic data processing (including peak fitting) *in situ* and time-resolved investigation of chemical reactions would be much more cumbersome and less efficient.

### 4.2. Automated calibration and data reduction   

The ability to script the calibration and reduction routines makes it possible to trigger fully automatic calibration and reduction of the two-dimensional diffraction data. This is important for the LDE facility, which at its full potential records hundreds of diffraction images at up to 20 different diffraction geometries during a measuring day (Murray *et al.*, 2017 ▸). Thus, the processed data are made automatically available as diffractograms for further structural refinement.

**Figure 6:**
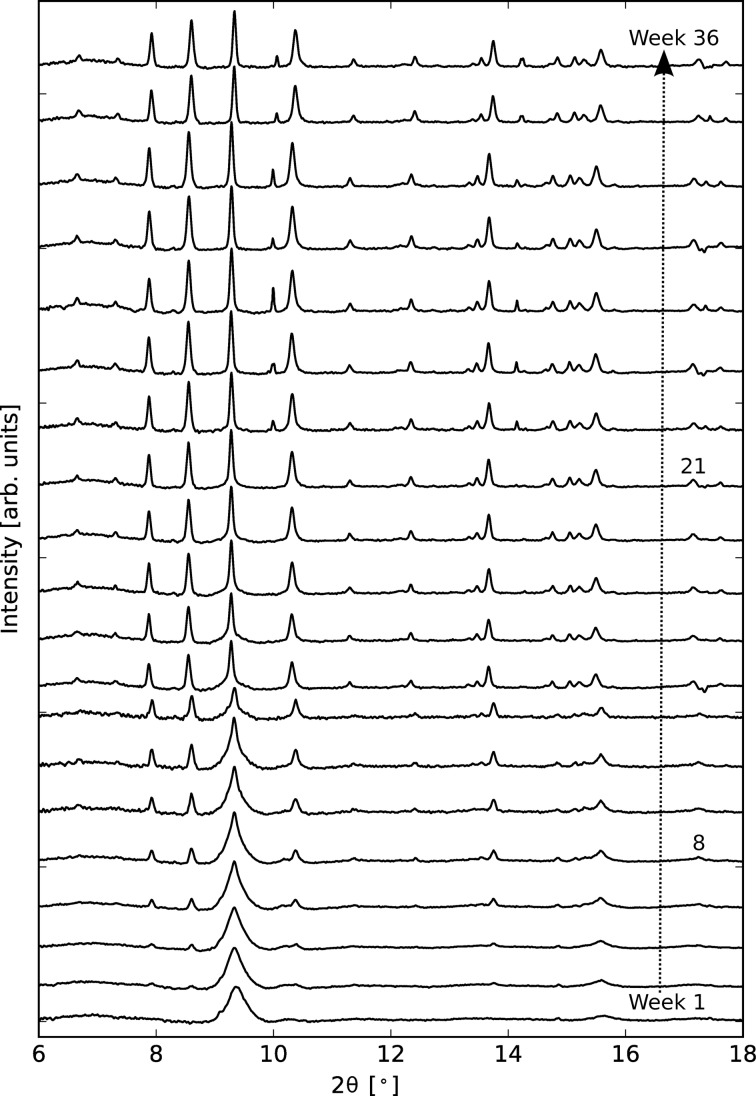
Selected powder patterns showing the evolution of initially amorphous calcium silicate exposed to atmospheric CO_2_ over a period of 36 weeks. The main conversion to crystalline calcium carbonate has almost finished by week eight but small changes are still occurring at week 21. Data were collected at 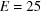
 keV. The diffractograms are vertically shifted with respect to the bottom pattern.

As an example for the automated conversion of a two-dimensional to a one-dimensional diffraction pattern the aging of initially amorphous calcium silicate (CaSiO_3_), while exposed to atmospheric CO_2_, is shown. Fig. 6 ▸ displays only a selection of the diffraction patterns that were recorded once a week for more than nine months. The conversion of amorphous CaSiO_3_ to crystalline calcium carbonate (CaCO_3_) over time becomes evident by the formation of Bragg peaks identifying two polymorphs of CaCO_3_: calcite and vaterite. The main conversion appears to be complete after eight weeks of exposure; however, the appearance of additional Bragg peaks at week 21 indicates that the material structure is still evolving. Understanding such (slow) processes responsible for the carbonation of calcium silicates is of relevance to the formation of carbonate minerals in astrophysical environments and also has potential applications in the sequestration of industrial CO_2_. More examples to show the effectiveness of the reduction package can be found in the paper by Murray *et al.* (2017 ▸).

### 4.3. SAXS grid scans   

SAXS is a versatile technique, capable of characterizing the shape and structure of partially ordered materials. Aside from carefully chosen intensity corrections (Pauw, 2013 ▸), SAXS experiments can have quite different processing requirements depending on whether it is the macromolecular shape or orientation of the sample that is being studied. A common approach for investigating heterogeneous orientated structures is to perform two-dimensional grid scans and to generate a map representing the strength and direction of the orientation. For two-dimensional detectors the size of the raw data files can be large, and although it is possible to get some indication of the orientation from the raw data, it is a slow and laborious process.

**Figure 7:**
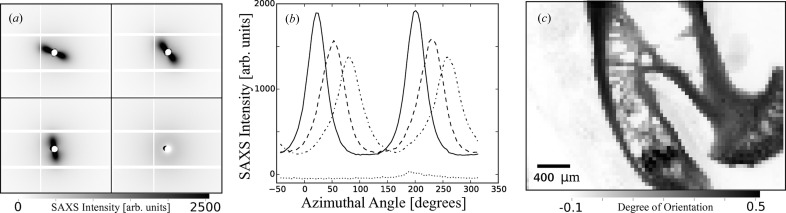
(*a*) Sections of four raw SAXS images obtained from a large grid scan across a bone sample. After various corrections have been applied to the raw images, each of them is mapped on azimuthal angle *versus q* cake plots. Then the integration over *q* yields (*b*) the four intensity *versus* azimuthal angle curves. The positions of the peak maxima in each curve reflect the intensity distribution in each of the raw datasets. The integration of all of these curves in the grid scan eventually leads to (*c*) a map that shows the degree of orientation of the scanned area of the sample.

The raw data for SAXS grid scans of, for example, a bone sample performed at I22 are saved as NeXus files, which allows the dataset dimension to reflect the dimensionality of the scan (in this case a four-dimensional dataset: two-dimensional scans of two-dimensional images). The mineral scatter from a selection of detector images from three regions of the sample which contain strong orientation and one that exhibits no orientation is shown in Fig. 7 ▸(*a*). The process chain contained, amongst others, modules to integrate the detector images to intensity *versus* azimuthal angle over the mineral scatter region (Fig. 7 ▸ *b*), and then this integrated pattern was used to calculate the metrics describing the orientation of the sample. Fig. 7 ▸(*c*) shows a measure of the proportion of X-rays scattered from parts of the sample with orientation relative to the total scatter, *i.e.* a measure of the degree of orientation of the sample. Since the processing maintains the rank of the raw data and propagates axes with the data, the output NeXus files contain these metrics as two-dimensional datasets with the sample stage axes, which allows the SAXS measurements to be related back to the sample.

## 5. Conclusion   

A software package for the calibration of the diffraction geometry of experiments using two-dimensional detectors and the processing of two-dimensional data has been described. It provides an accurate and reliable calibration even for high energies and detectors at large tilt angles, as well as the ability to configure processing pipelines for repetitive tasks to be executed automatically. Moreover, the output files maintain the data provenance, which not only gives the ability to reprocess data in a different manner at a later stage but also guarantees transparency of the data treatment. The *DAWN 2* package enables new types of data collection, as highlighted by the automated calibration and processing of long-duration experiments and the ‘on-the-fly’ data reduction which is highly beneficial for *in situ* time-resolved measurements. Fairly complex processing tasks on large multi-dimensional data sets, generated for example by grid scans in SAXS experiments, are easy to include in the processing pipeline, as are customized processing steps in the form of Python scripts. Owing to its ability to read a wide variety of data formats, data obtained at other facilities or at the home laboratory can be treated as well.

## Footnotes

1
*DAWN 2.4.0* is freely available for download from http://dawnsci.org. It runs on Windows, Linux and MacOS and requires a 64 bit processor and a minimum of 1 GB available RAM.

## Appendix A Scripting

In the following an example of a script for calculating a Kratky data set from intensity *versus q* data is given. Although this example is trivial, it exemplifies how straightforward it is to use the rich libraries available in Python to develop and incorporate new processing steps into the processing chain. It shows how the data are automatically pushed into Python and how Python returns them to the processing pipeline.

import numpy as np

def run(xaxis,data, **kwargs):

   
kratky = np.power(xaxis,2)*data

   
script_outputs = {"data" : kratky}

   
script_outputs["xaxis"] = xaxis

   return script_outputs

## Appendix B Data formats supported by *DAWN*

Although the software was built specifically for use with data collected by beamlines at DLS, the features described in this article are available to use on any data format *DAWN* is able to load. Currently supported data formats include HDF5 files (and derived formats such as NeXus, NetCDFv4, Matlab v7.3), Tiff, EDF, Mar (.mar3450, .mccd), CBF and *Fit2D* (.f2d), as well as JPG, PNG and a variety of ASCII formats.
